# Development of In Vitro Dry Eye Models to Study Proliferative and Anti-Inflammatory Effects of Allogeneic Serum Eye Drops

**DOI:** 10.3390/ijms24021567

**Published:** 2023-01-13

**Authors:** Silja Voß, Till Behrmann, Stephan Reichl

**Affiliations:** Institut für Pharmazeutische Technologie und Biopharmazie, Mendelssohnstraße 1, Technische Universität Braunschweig, 38106 Braunschweig, Germany

**Keywords:** in vitro model, dry eye, allogeneic serum, wound healing, matrix metalloprotease 9, design of experiments, dynamic flow system

## Abstract

This study aimed to develop valid in vitro models for preclinical evaluation of proliferative and anti-inflammatory effects of human allogeneic serum eye drops for dry eye disease (DED) treatment. A DED wound healing model was developed by analyzing the influence of coating and serum concentrations on human corneal epithelial (HCE-T) wound closure. Further, intralaboratory variance, freeze–thaw cycle effects, donor variability and stability assays were conducted. Interleukin-1β (IL-1β) and tumor necrosis factor α (TNFα) were used to induce the gene expression of matrix metalloproteinase 9 (MMP9), cyclooxygenase 2 (COX2), transforming growth factor-β (TGFβ) and IL-1β. MMP9 induction was optimized using a design-of-experiments (DoE) approach and applied to examine serum under static and dynamic conditions. MMP9 protein expression was analyzed by ELISA. The DED wound healing model detected proliferative effects of serum down to 1% with a small intralaboratory variance. Serum stability was shown over six months, donor variance could be detected, and freeze–thaw cycle effects did not affect wound closure. Serum decreased MMP9 expression on the gene and protein levels. The induction method was successfully optimized using DoE modeling and transferred to a dynamic setting mimicking tear film fluidics. The DED wound healing and inflammatory DED model present useful in vitro models for the preclinical evaluation of allogeneic serum eye drops without the use of animal experiments.

## 1. Introduction

Dry eye disease (DED) is defined as a multifactorial disease with an unstable tear film, with reduced tear film homeostasis followed by inflammation and ocular surface deterioration, including neurosensory aberrations [1]. The quality of life of DED patients is reduced by the discomfort caused by sensations such as itching, burning, irritation and visual disturbance, leading to restrictions in daily activities [2,3]. DED plays an increasingly important role in aging societies, as its prevalence rises with age [3].

DED therapy options are chosen depending on the severity of the disease. Jones et al. [4] described four different therapy stages. In stage one and stage two, artificial tears without preservatives are recommended to moisturize the ocular surface. In stage two, topical medication may be used, for example, topical glucocorticosteroids, lymphocyte function-associated antigen-1 antagonists or immunomodulators such as cyclosporin. In stage three, the use of therapeutic contact lenses or serum eye drops is recommended. In stage four, surgical operations are the optimal choice.

Autologous serum eye drops are a useful therapy option for stage three DED. They are produced using the patient’s own blood and are frequently examined in clinical studies [5,6,7,8,9,10]. Autologous serum eye drops resemble the tear film more completely than artificial tears, as they possess physiological similarities to the tear film such as comparable pH and osmolarity [11,12,13]. They also contain growth factors and proteins that support wound healing at the ocular surface [8,14,15,16,17]. Thus, in addition to moisturizing the ocular surface, serum eye drops show wound healing and anti-inflammatory effects in in vitro studies [18,19]. Furthermore, autologous serum eye drops did not reveal any significant side effects during treatment compared to other anti-inflammatory therapy options [20,21].

Although autologous serum proved to be a promising treatment, there are some limitations to this therapy option. Not all patients in need of serum treatment are fit to donate their own blood for therapy due to cardiovascular or cerebrovascular diseases, infections or the patient’s age [21,22]. Hence, allogeneic serum eye drops, which are produced from blood donations, could be a useful alternative. Allogeneic serum ameliorated the clinical symptoms of DED similarly to autologous eye drops in clinical studies [20,21,23,24]. Allogeneic and autologous serum eye drops therefore present a complementary approach to DED treatment.

Before allogeneic serum eye drops can be used as a licensed medicine on the market, preclinical tests must be conducted to prove their wound healing and anti-inflammatory effects. A wide range of preclinical models have been developed over the years. They can be divided into animal-based or in vitro cell culture models. Animal models are widely used in the development of new drugs [25]. However, they lack transferability to human tissue, possess ethical constraints and are expensive. In vitro cell culture assays are easily transferable due to human tissue usage, have few ethical constraints and are easier to reproduce [26]. Therefore, in vitro cell culture models play an important part in preclinical drug development.

Human in vitro cell culture models can be classified using different criteria. There are 2D, 3D and dynamic cell culture models using either immortalized cell lines [27,28,29] or primary cells [30]. Monolayer 2D cell culture models are time efficient in cultivation and are an adequate choice for large sample sizes. Three-dimensional cell culture models better mimic the in vivo environment, can foster cell interactions and develop organotypic properties [31]. Dynamic cell culture models are microfluidic systems creating flow conditions to imitate organotypic fluidics, such as the tear film [32]. Mimicking tear film fluidics at the ocular surface helps to create in vivo like surroundings with the advantages of an in vitro model [32,33].

A specific type of cell culture model is a wound healing model. In vitro wound healing models represent a commonly used approach to study the proliferative and migratory properties of substances [34,35,36,37,38]. They are a common method used as a wound creation surrogate in various diseases, including DED [35,39,40]. However, there are only a few studies exploring the variability of the assay [41]. Therefore, a study focusing on the repeatability and intralaboratory variance of wound healing models as well as reproducible preclinical tests of allogeneic serum eye drops should be conducted.

There are various inflammatory models to imitate DED pathophysiology in cell culture models. Desiccating stress models create dehydration of the cell surface area by adapting the culture conditions [42,43]. Furthermore, hyperosmolar induction models use hyperosmolar medium to achieve DED under similar conditions [44,45,46]. Additionally, the use of cytokines to induce inflammation has previously been described [30,47]. These studies all followed trial and error approaches to find the optimal induction conditions. Unlike trial and error approaches, design-of-experiments (DoE) models assess research questions in a more systematic, integral and faster way [48]. For qPCR methods, there have been a few DoE models that focused on optimizing the product yield and probe quantity or performed a computer-based simulation of the qPCR method [49,50,51]. A study looking at the optimization protocol of induction conditions has not yet been conducted.

The present study established in vitro DED models to show the wound healing and anti-inflammatory potential of allogeneic serum eye drops for preclinical tests. The first part of this study focuses on the development of a DED wound healing model and on valid, reproducible preclinical wound healing tests of allogeneic serum. In the second part of the study, a DoE method was developed to distinguish the optimal induction conditions for an inflammatory DED model. This model was transferred to dynamic conditions in a further optimization step to gain easily transferable data with in vivo like culture conditions.

## 2. Results

### 2.1. DED Wound Healing Model

#### 2.1.1. Assay Development

**Figure 1:**
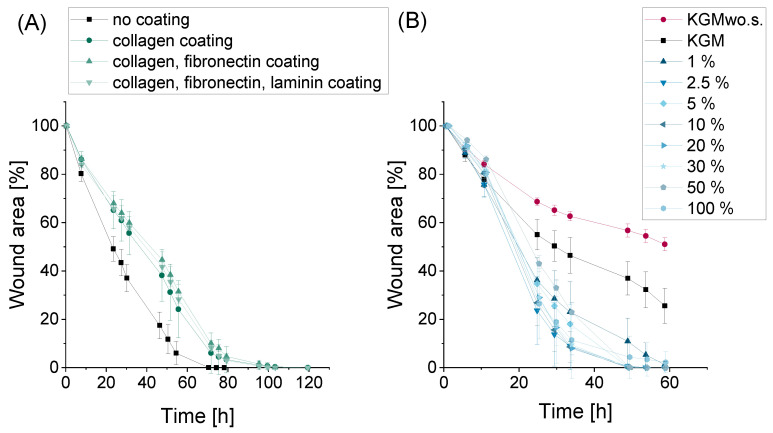
DED wound healing model: (**A**) influence of coating material combinations with collagen, fibronectin and laminin on wound closure in keratinocyte growth medium (KGM); 3–4 wells per sample were analyzed (n_well_ = 3–4); 6 pictures of the wound area were taken per well (n_picture_ = 6), mean ± standard deviation (SD); (**B**) different serum concentrations in keratinocyte growth medium without supplements (KGMwo.s.) (n_well_ = 3–4, n_picture_ = 6, mean ± SD).

The first part of the project aimed to develop a reproducible DED wound healing model to study the proliferative and migratory effects of allogeneic serum in preclinical applications. Therefore, three different coating combinations with collagen, fibronectin and laminin were compared to uncoated wells to examine their effect on HCE-T-cell wound closure. All tested combinations reduced the wound area in HCE-T cells (Figure 1A). As the examined coating materials did not have a positive influence on the wound closure rate, the following studies were carried out with uncoated wells. Furthermore, a wide range of serum concentrations was analyzed to show differences in serum samples in preclinical studies for their wound-healing potential and to find an effective concentration. All serum concentrations from 1% to 100% accelerated the wound closure rate of HCE-T cells (Figure 1B). The wound-healing effect was similar at all depicted concentrations. Hence, the serum samples did not show a concentration-dependent effect. Therefore, small serum concentrations of 1% or 2.5% were used to study serum effects in the present study.

**Figure 2:**
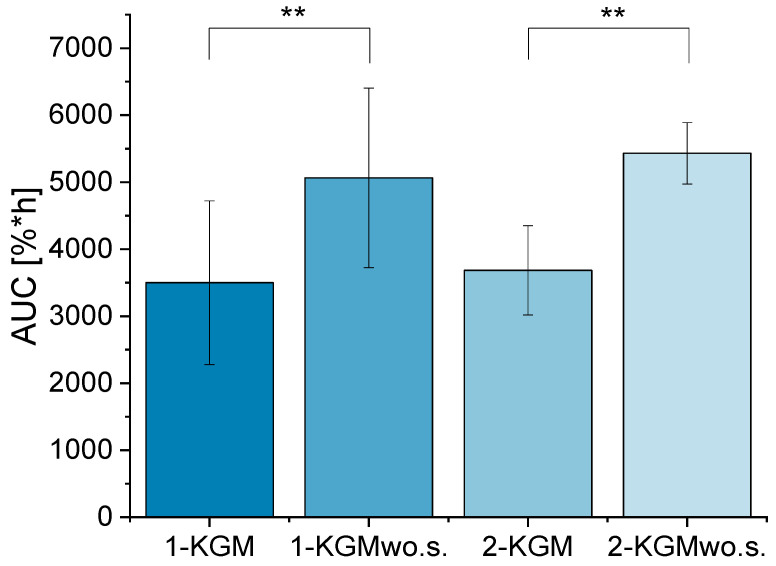
AUC of KGM and KGMwo.s. of researcher 1 and 2; researcher 1 analyzed 33-34 wells per sample (n_well_ = 33–34), 6 pictures of the wound area were taken per well (n_picture_ = 6); researcher 2: n_well_ = 4, n_picture_ = 5–6; mean ± SD, an independent *t* test was conducted with ** *p* ≤ 0.01.

**Figure 3:**
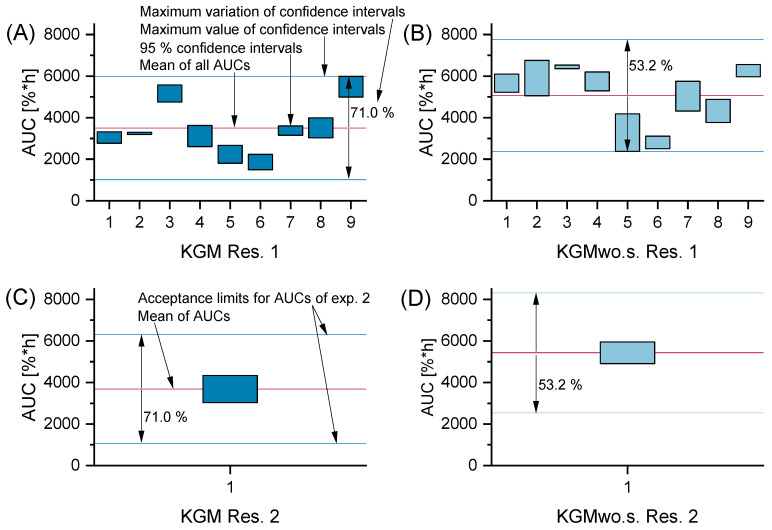
Intralaboratory variance of the DED wound healing model with an equivalence test; (**A**,**B**) the maximal variation of the confidence intervals of researcher 1 (Res. 1) was used to determine a relative variability of 71.0% for KGM and 53.2% for KGMwo.s.; nine independent experiments were conducted; Res. 1 analyzed 33–34 wells per sample in those nine experiments (n_well Res. 1_ = 33–34); (**C**,**D**) the same relative variability was applied to the mean of the AUCs of KGM and KGMwo.s. of researcher 2 (Res. 2) to define the cutoff values for the confidence intervals of Res. 2 to be considered tolerable (n_well Res. 2_ = 4).

An independent *t* test of the control samples was conducted to study the functionality of the DED wound healing model. The independent *t* test results showed a significant difference in the area under the curve (AUC) of the positive and negative controls in both researchers (Figure 2). Furthermore, the equivalence test examined the intralaboratory variance between the two different researchers. The limits were based on the greatest deviation of the confidence intervals of the AUCs from the positive and negative controls from the researcher one. It was calculated as a percentage relative to the mean value of the AUC. Then, the limits of researcher two were set based on the same percentage-based deviation. The 95% confidence intervals of researcher two of the control samples fitted into the set limits of researcher one (Figure 3). Thus, the AUC of the control samples showed a small interpersonal variance, suggesting that the DED wound healing model is easily transferable to another researcher in the same laboratory.

#### 2.1.2. Preclinical Applications

**Figure 4:**
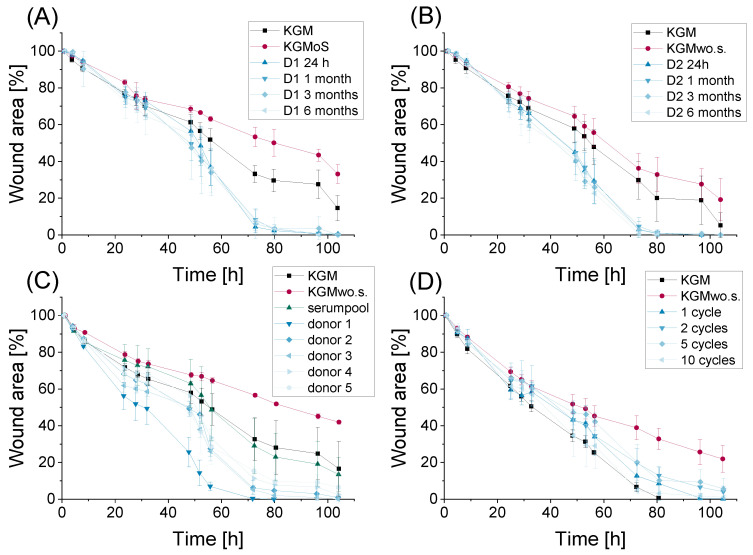
(**A**,**B**) Stability assay of 2.5% serum from donors 1 and 2 (D1, D2) after storage at −20 °C for 24 h, 1 month, 3 months and 6 months; 3 wells per sample were analyzed (n_well_ = 3); 6 pictures of the wound area were taken per well (n_picture_ = 6), mean ± SD; (**C**) donor variability of serum 1% (n_Well_ = 4, n_picture_ = 6, mean ± SD); (**D**) influence of freeze–thaw cycled serum 2.5% on wound closure of HCE-T cells in KGMwo.s. medium (n_well_ = 3–4, n_picture_ = 6, mean ± SD).

After developing the DED wound healing model, it was used for preclinical applications. First, a stability assay of serum samples from two different donors was conducted. The serum samples of both donors showed an accelerating effect on wound closure at all analyzed storage times (Figure 4A,B). Moreover, this serum effect was visible in all depicted samples for up to six months of serum storage at −20 °C.

Furthermore, the variability of donor samples was analyzed. All examined donors showed a promising effect in the DED wound healing model at the analyzed serum concentration of 1% (Figure 4C). Additionally, the model was able to show differences in donor samples, as the HCE-T cells treated with serum from donor one demonstrated a faster wound closure compared to that of the other donors.

The influence of different freeze–thaw cycles on serum stability was also tested. All depicted freeze–thaw cycles had no negative influence on the wound closure rate of HCE-T cells in KGMwo.s. medium (Figure 4D). Their wound closure was comparable to the wound closure of the positive control KGM medium. Hence, the wound healing model was able to distinguish between the effects of different serum samples in preclinical applications.

### 2.2. Inflammatory DED Model

#### 2.2.1. Static Inflammatory DED Model

**Figure 5:**
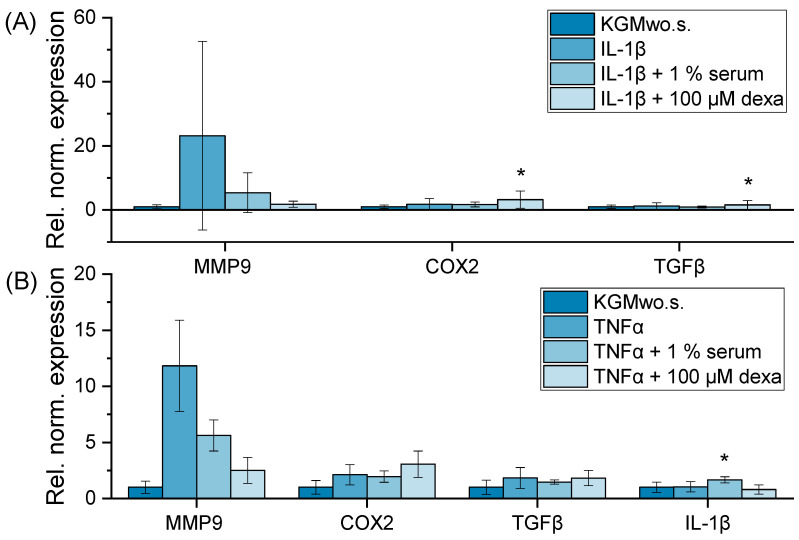
Relative normalized gene expression of MMP9, COX2, TGFβ and IL-1β in HCE-T cells; 24 h of cultivation with IL-1β (**A**) or TNFα (**B**) (10 ng/mL) in KGMwo.s. medium; 3–6 wells per sample were analyzed (n_well_ = 3–6); 2–3 technical replicates were conducted per well (n_technical replicate_ = 2–3); mean ± SD, one-way ANOVA with * *p* ≤ 0.05.

The second part of the project aimed to develop a reproducible DED inflammatory model to study the anti-inflammatory effects of allogeneic serum in preclinical applications. To find suitable inflammatory markers, IL-1β and TNFα inductions were tested for their effect on the expression of different inflammatory genes. MMP9 was the only gene that showed significant amplification after adding TNFα or IL-1β (Figure 5). COX2, TGFβ and IL-1β did not show increased gene expression after induction. Both serum 1% and the positive control dexamethasone showed a visible reduction in MMP9 expression (Figure 5). Hence, MMP9 was used as an inflammatory marker in the following optimization process.

##### Design of MMP9 Gene Expression Experiments

**Figure 6:**
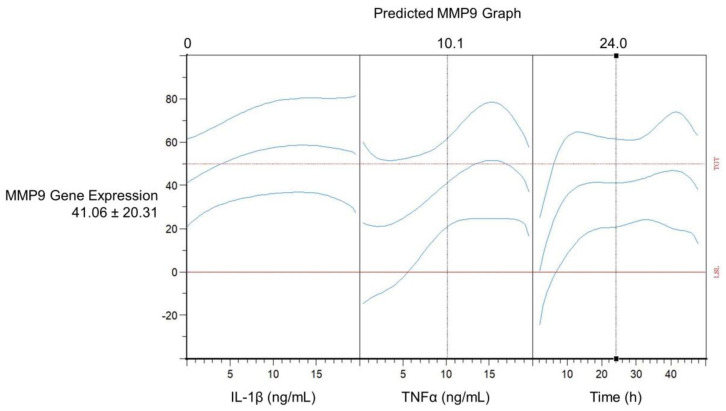
Predicted response graph in Cornerstone: IL-1β concentration (ng/mL), TNFα concentration (ng/mL) and time (h) are plotted against MMP9 expression and its 95% confidence intervals (blue graphs); the dotted red line shows the target value of the model (50).

With the help of the DoE studies, an optimized model of MMP9 induction was generated. Table 1 shows the coefficient table of the terms used in the model. The adjusted R-square of the model is 0.966. Figure 6 depicts the predicted response graph as shown in the Cornerstone software. The MMP9 expression of 41.06 ± 20.31 (Figure 6) was estimated by the model using the following settings: IL-1β concentration of 0 ng/mL, TNFα concentration of 10.1 ng/mL and 24.0 h induction time.

**Figure 7:**
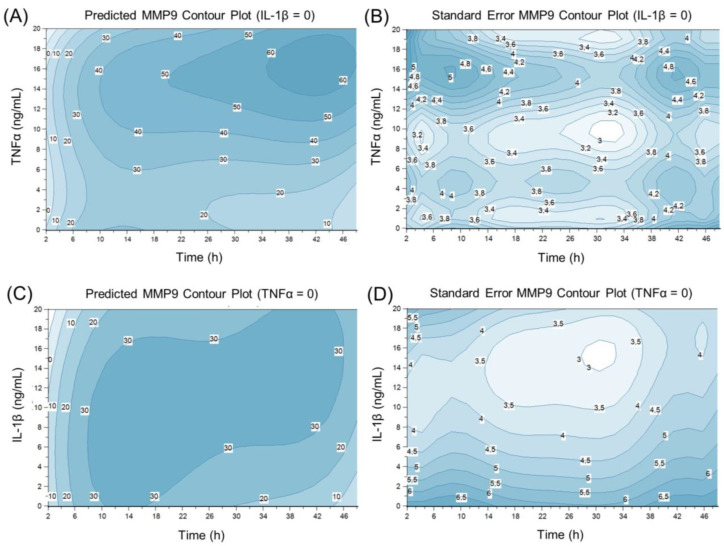
Contour plots of predicted MMP9 expression and standard error at single TNFα (**A**,**B**) or IL-1β (**C**,**D**) induction over time.

**Figure 8:**
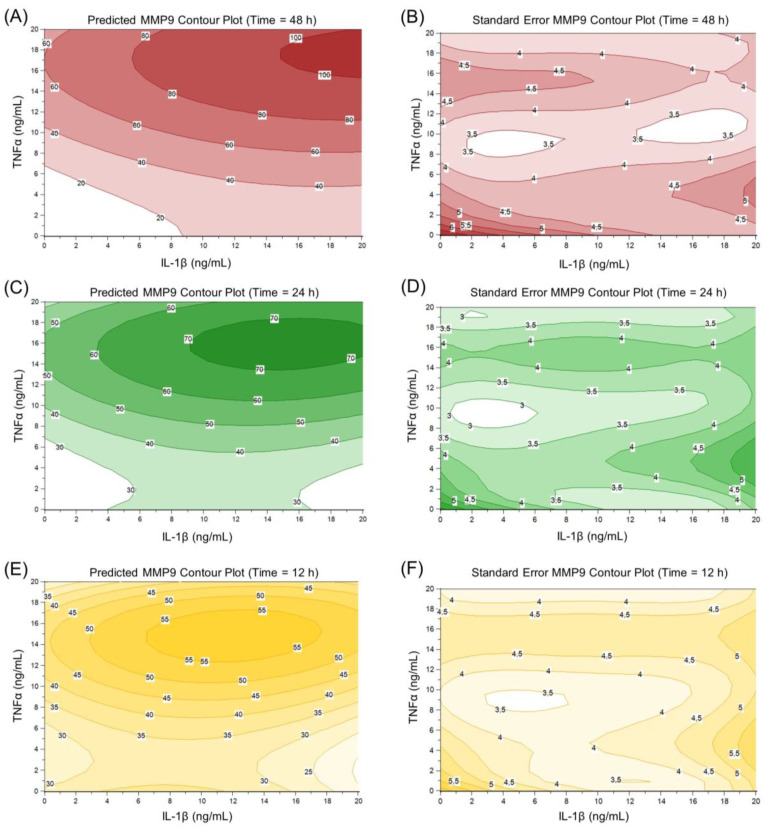
Contour plot of predicted MMP9 expression and standard error at 48 h (**A**,**B**), 24 h (**C**,**D**) and 12 h (**E**,**F**) depending on TNFα and IL-1β concentrations.

The predicted MMP9 expression depending on IL-1β and TNFα concentrations and the induction time in HCE-T cells is illustrated by contour plots of the relative normalized MMP9 gene expression and their standard error (Figure 7 and Figure 8). MMP9 expression increases with increasing induction time. Examining the separate induction of TNFα and IL-1β, TNFα seems to be the more potent inducer with the highest MMP9 gene expression of 60 between 36 and 48 induction hours (13-19 ng/mL TNFα) compared to a gene expression of 30 between 10 and 46 induction hours (1-20 ng/mL IL-1β) (Figure 7). The standard error of the estimated MMP9 expression rises at the edge of the model (Figure 7 and Figure 8). This higher standard error might arise because there are less data to underline the predicted MMP9 expression at the edge of the DoE model. Therefore, it seems favorable to use induction parameters in the middle of the DoE model. As the standard error is lower in this part of the DoE design, the results are easier to predict and are therefore more reliable. The use of one inducer at a time for sufficient MMP9 expression is inexpensive and more easily accessible. TNFα seems to be the better choice as a single inducer, as MMP9 expression is higher with TNFα than with IL-1β (Figure 7). Thus, a 24 h induction time and a TNFα concentration of 10 ng/mL were chosen for further experiments as these induction parameters resulted in a relatively high predicted gene expression of approximately 40 and a relatively stable and low standard error of approximately 3.2. 

##### Preclinical Serum Concentration Screening

**Figure 9:**
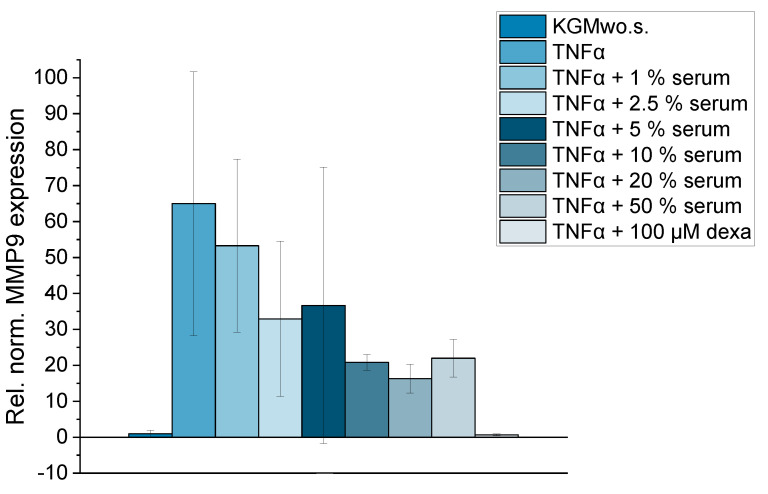
Relative normalized gene expression of MMP9 in HCE-T cells; 24 h of cultivation with TNFα (10 ng/mL) in KGMwo.s. medium with different serum concentrations: 1%, 2.5%, 5%, 10%, 20%, 50%; 3 wells per sample were analyzed (n_well_ = 3); 2–3 technical replicates were conducted per well (n_technical replicate_ = 2–3); mean ± SD.

The optimized DED inflammatory model was used to examine the anti-inflammatory potential of different serum concentrations. All analyzed serum concentrations reduced MMP9 gene expression after induction with TNFα (Figure 9). This effect increased with higher serum levels, apart from 50% serum, which reduced MMP9 gene expression slightly less than the 20% sample. Hence, the DED inflammatory model was able to illustrate anti-inflammatory serum effects on the gene expression of MMP9.

#### 2.2.2. Dynamic Inflammatory DED Model

**Figure 10:**
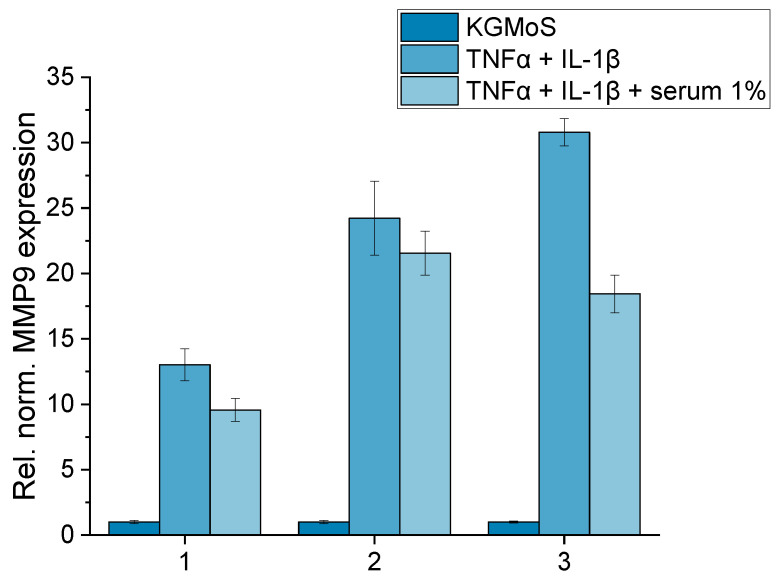
HCE-T cells were cultivated with TNFα (10 ng/mL) and IL-1β (10 ng/mL) for 72 h; serum was added under dynamic conditions in the Ocular DynaMiTES system with a turnover rate of 8.3% and a flow rate of 75 µL/min for 180 min; gene expression of MMP9 is depicted; the numbers 1–3 on the abscissa depict three independent experiments with 1 well per sample (n_well_ = 1) and 2–3 technical replicates per well (n_technical replicate_ = 2–3); mean ± SD of technical replicates.

The static DED inflammatory model was adapted to dynamic conditions to obtain a model that is closer to in vivo microfluidic conditions. HCE-T cells showed a reduction in MMP9 gene expression after serum addition in the dynamic flow system (Figure 10). The 72 h induction with cytokines TNFα and IL-1β was able to raise MMP9 expression up to 30.79. The cells responded to the short period of the DynaMiTES incubation, in which serum was added, with a reduction in MMP9 expression. The addition of serum under dynamic conditions reduced this expression in all three independent experiments (Figure 10). Hence, the anti-inflammatory effect of allogeneic serum eye drops in the context of MMP9 expression was successfully displayed under dynamic conditions.

#### 2.2.3. Inflammatory DED Protein Expression Model

**Figure 11:**
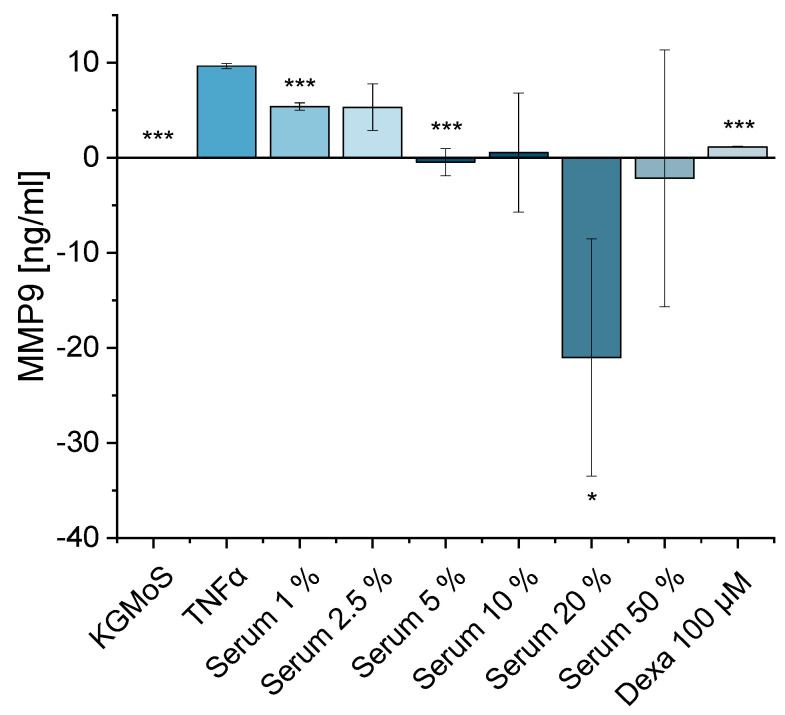
Protein expression of MMP9 in HCE-T cells; 24 h of cultivation with TNFα (10 ng/mL) in KGMwo.s. medium with different serum concentrations: 1%, 2.5%, 5%, 10%, 20%, 50%; 100 µM dexamethasone served as a positive control, KGMwo.s. medium as a negative control; 3 wells per sample were analyzed (n_well_ = 3); 2 technical replicates were conducted per well (n_technical replicate_ = 2); mean ± SD, one-way ANOVA with * *p* ≤ 0.05 and *** *p* ≤ 0.001.

Finally, the anti-inflammatory effect of serum was tested in an MMP9 protein expression model. MMP9 protein expression was analyzed after TNFα induction for 24 h with the addition of different serum concentrations. TNFα induction increased MMP9 expression at the protein level (Figure 11). As the cell culture supernatants were analyzed, the MMP9 content from the added serum had to be considered. Human serum samples themselves possess MMP9 [52,53] and can obscure the serum effect on the cells. Thus, the effect of MMP9 in pure serum samples was subtracted as a positive control from the data. The HCE-T cells showed a concentration-dependent reduction in MMP9 expression (Figure 11). All depicted concentrations showed a concentration-dependent decrease in MMP9 levels in the cell culture supernatant. The negative MMP9 expression after the addition of 20% and 50% serum might be due to high MMP9 expression in the serum positive control. The results of 1%, 5% and 20% and the dexamethasone control sample were significantly different from those of the TNFα-treated cells. The other samples were not significantly different from the TNFα-treated cells due to their high standard deviation (Figure 11). Thus, the trend of the anti-inflammatory effect of serum on MMP9 expression was confirmed at the protein level.

## 3. Discussion

### 3.1. DED Wound Healing Model

The aim of the development of the DED wound healing model was to obtain a reproducible method to study the wound-healing effects of allogeneic serum eye drops for preclinical tests. Overall, an assay optimization experiment with coating materials had no positive impact on wound closure. Moreover, the wound healing rate of HCE-T cells was accelerated similarly by all analyzed serum concentrations. The model was examined for reproducibility and showed small intralaboratory variance. During preclinical testing, the DED wound healing model was able to distinguish between different serum samples in their wound-healing potential and to evaluate the stability, donor variability and freeze–thaw cycle effects of serum.

The wound-healing rate was reduced by the coating material. This reduction could be due to overlaying edges of the coating material in the scratch area, which might have made it more difficult for the cells to grow together. Hence, the scratch creation in the cell monolayer seemed to remove the coating materials from the cell dish surface. This has also been described in previous research [54]. Therefore, it is more useful to implement a scratch protocol without the use of extracellular matrix coating in the classical scratch 2D protocol. Another option is the use of different wound creation techniques, such as inserts for cell growth exclusions in defined areas (Ibidi, Gräfelfing, Germany). However, they have the disadvantage of no mechanical wound creation. In the application of this particular study, the extracellular matrix coating had no beneficial effect on wound closure.

The wound-healing effect of serum is in line with previous in vitro studies. In a study from Wu et al. [18] positive effects of 5% to 30% human serum on wound healing of HCE-T cells were shown. Furthermore, a study by Robciuc et al. [35] showed a wound-healing effect of 2% fetal bovine serum in a scratch assay in HCE-T cells. Thus, the current study’s data align with the results from other in vitro studies of serum in wound healing assays. Therefore, this DED wound healing model is able to depict the wound-healing capacities of different serum concentrations for preclinical studies.

Pipette tips are often used as a scratch creation technique [38,39,40,54]. Apart from wound creation with pipette tips, magnet-based scratch creation techniques [41], circular punch injuries [36] and the use of special inserts to grow cell-free areas [55] are described in the literature. Magnets and inserts possessed less variation in the scratch width and the straightness of the gap width compared to pipette tips [41]. Nevertheless, this study showed that pipette tips could still produce reproducible wound areas with a low intralaboratory variance. This was shown by similar results of positive and negative controls in the wound healing model when carried out by two different researchers in the same laboratory. Thus, a reproducible DED wound healing model was created, which was thereafter used in several preclinical examinations to test its functionality. Further studies should focus on interlaboratory variance and the influence of scratch width differences on the repeatability of the wound healing model.

The storage stability of up to six months for serum eye drops has been previously described. Fischer et al. [56] showed that serum maintained its wound-healing potential and showed no significant reduction in growth factor amounts after storage for six months at −20 °C. These data are in line with the findings of the present study showing serum stability up to six months at −20 °C. Additionally, the present study showed stability for a serum concentration of 2.5%. This low concentration is useful to detect differences in the wound-healing potential of varying serum qualities.

Furthermore, the analysis of donor variability showed the advantage of low serum concentrations in preclinical studies. The present study was able to show differences in the wound-healing potential of different donors, which was possibly due to the low serum concentration of 1% in the assay. Hence, the DED wound healing model can be used to identify variations in the serum effectiveness of different donors or serum batches.

There has been research on serum proteins and their stability in freeze–thaw cycles. C-reactive protein levels were found to be stable for up to ten freeze–thaw cycles in serum samples [57]. In another study, freeze–thaw cycles did not affect the serum levels of several endocrine markers, such as cortisol binding globulin and glucagon, with the exception of plasma renin and adrenocorticotropic hormone [58]. These data are in line with the data of this study, as there was no negative effect of freeze–thaw cycles on wound closure rates. However, this lack of effect of freeze–thaw cycles is not found with all markers. Lipid metabolites such as arachidonic acid were significantly decreased in serum and plasma levels after ten freeze–thaw cycles [59]. The findings of the present study suggest that serum ingredients with wound-healing potential are not inactivated by up to ten freeze–thaw cycles. Nevertheless, this does not mean that all active substances are stable during repeated freezing and thawing, as the study of Ishikawa et al. [59] has shown. Therefore, the number of freeze–thaw cycles for serum sample preparation should be kept to a minimum. Furthermore, it was possible to show the effects of freeze–thaw cycles on the wound closure potential of serum samples in this preclinical application.

Considering all data, this model can be used to study the wound-healing potential of allogeneic serum eye drops in diverse preclinical applications. It presents a useful method to find effective serum concentrations and to obtain valuable information on the stability and donor variability of serum samples in preclinical tests without the use of animal models. Hence, this wound healing model might be used to study other DED-related drug candidates in future preclinical applications.

### 3.2. Inflammatory DED Model

The inflammatory DED model successfully induced MMP9 expression with TNFα or IL-1β induction. The optimized protocol showed the anti-inflammatory capacities of human allogeneic serum eye drops under static and dynamic conditions in preclinical settings.

TNFα and IL-1β were previously used inflammatory inducers in preclinical studies [47,60,61], including in vitro dry eye models [62]. None of these models tested the induction method in advance with a DoE model. A DoE MMP9 expression model of HCE-T cells was successfully developed in the present study. Compared to previous trial and error models, the present DoE model presents a more systematic and time-saving approach to find optimal induction parameters. The model predictions still possess certain deviations due to the existing uncertainties in the form of confidence intervals. These factors must be considered when interpreting the results. Overall, this model helps to verify and select the best induction method and may be used for similar experimental design issues in the future.

MMP9 plays an important role in the pathophysiology of DED [1,63]. Furthermore, MMP9 is used as a clinical marker for dry eye disease in the tear film of patients [64]. The MMP9 level in the tear film of DED patients was found to be higher than that in healthy tear films, and it correlated with DED diagnostic criteria such as the Schirmer test or tear break-up time [65,66,67]. Hence, MMP9 is a useful marker in both preclinical and clinical contexts and was used in this model as an inflammatory marker.

The analyzed MMP9 expression in HCE-T cells is in line with previous studies showing expression of this marker in HCE-T cells [27]. The reduction of MMP9 gene expression by serum was previously described in RAW 264.7 cells with the use of mouse serum and fetal bovine serum [68]. Lee et al. [68] showed an MMP9-reducing effect of mouse serum in a concentration-dependent manner and suggested an inhibitory effect independent of IFN-γ activity. The results of the present study are in line with this study, as serum could reduce MMP9 expression in a concentration-dependent manner in HCE-T cells. The effect of human allogeneic serum on the in vitro MMP9 protein expression in corneal epithelial cells has not been described previously. The reduction in MMP9 by serum was confirmed at the gene and protein levels in the present study. Therefore, MMP9 was successfully used to demonstrate the anti-inflammatory behavior of allogeneic serum in a DED cell culture model.

In addition to MMP9, the present study examined other inflammatory markers and the serum effect on their gene expression. COX2, TGFβ and IL-1β were not induced by IL-1β or TNFα. This may be due to a lack of expression of these markers in HCE-T cells or inflammatory cascades where the inducers do not affect COX2, TGFβ and IL-1β expression. Hence, COX2, TGFβ and IL-1β do not present useful DED markers in this specific inflammatory DED model.

There are several dynamic DED models in the literature. One study analyzed the influence of shear stress on human corneal cells [69], and another study showed that dynamic cultivation enhances cellular metabolism [70]. Neither of these models analyzed the anti-inflammatory serum effect on HCE-T cells in a dynamic environment. Therefore, this study analyzed the anti-inflammatory potential of allogeneic serum eye drops in a dynamic, more in vivo like environment for preclinical applications. Moreover, it helps in making hypotheses on the anti-inflammatory effect of substances in an early development state and in determining interesting drug candidates for further research. However, the developed dynamic inflammatory DED model possesses some limitations. The model is bound to an artificial in vitro environment. Although the dynamic system mimics the dynamic environment at the ocular surface, it does not compensate for true in vivo conditions in the eye. Therefore, in addition to the present study, in vivo studies to test the anti-inflammatory potential of allogeneic serum should be conducted in the future.

## 4. Materials and Methods

### 4.1. Materials

SYBR Green Supermix, iScript gDNA Clear cDNA Synthesis Kit, Hard-Shell 96-Well PCR Plates and Microseal B PCR Plate Sealing Film were purchased from Bio-Rad (Hercules, CA, USA). Six-well plates and Transwell^®^ inserts with polycarbonate membranes (1.12 cm^2^, 3.0 µm pore size) were procured from Corning Incorporated—Life Sciences (Corning, NY, USA). Pipette tips were purchased from Eppendorf (Hamburg, Germany). Isopropanol was obtained from CVH Chemie Vertrieb GmbH & Co Hannover KG (Hannover, Germany). KGM™ Keratinocyte Growth Medium BulletKit™ was obtained from Lonza (Basel, Switzerland). Twenty-four-well plates were purchased from TPP (Trasadingen, Switzerland). Laminin and IL-1β were purchased from Sigma–Aldrich by Merck (Darmstadt, Germany). PBS was procured from MP Biomedicals (Irvine, CA, USA). A mycoplasma test kit was obtained from PromoCell (Heidelberg, Germany). Human MMP-9 DuoSet ELISA, DuoSet ELISA Ancillary Reagent Kit 2 and normal goat serum were acquired from R&D Systems (Minneapolis, MN, USA). Sodium chloride and calcium chloride dihydrate were procured from Roth (Karlsruhe, Germany). RNAse-free pipette tips and tissue culture flasks were acquired from Sarstedt (Nümbrecht, Germany). Allogeneic human serum was kindly provided by SerEye GmbH (Göttingen, Germany). Fibronectin, chloroform, ethanol, DEPC water, TRIzol reagent and calcium chloride were purchased from Thermo Fisher Scientific (Waltham, MA, USA). Potassium chloride was acquired from Acros Organics by Thermo Fisher Scientific (Waltham, MA, USA). TNFα, trypsin and soybean trypsin inhibitor were acquired from Gibco by Thermo Fisher Scientific (Waltham, MA, USA). Primers were obtained from Invitrogen by Thermo Fisher Scientific (Waltham, MA, USA). Dexamethasone was kindly provided by Ursapharm (Saarbrücken, Germany).

### 4.2. Cultivation of HCE-T Cells

Human corneal epithelial (HCE-T) cells are an SV40-immortalized, genetically modified, adherent cell line. Primary corneal epithelial cells were isolated from a 49-year-old woman and then transfected with an SV40-adenovirus vector to obtain an immortalized cell line by Araki-Sasaki et al. [71]. HCE-T cells were purchased from RIKEN Cell Bank (Tsukuba, Japan). HCE-T cells were cultured in KGM medium under standardized conditions (37 °C, 5% carbon dioxide atmosphere). KGM medium consisted of keratinocyte basal medium and KGM™ Keratinocyte Growth Medium SingleQuots™ Supplements and Growth Factors containing bovine pituitary extract, human epidermal growth factor, insulin, hydrocortisone, gentamicin sulfate and amphotericin B. Additionally, calcium chloride solution was added to the medium up to a final concentration of 0.5 mM. The KGM medium was changed three times per week, and the cells were split once per week using trypsin and soybean trypsin inhibitor. HCE-T cells were regularly tested for mycoplasma contamination.

### 4.3. DED Wound Healing Model

For the wound healing model, HCE-T cells were seeded into 24-well plates (90,000 cells per well) one week before starting the wound healing assay. The wound was created by using a 1000 µL pipette tip to scratch the well plate from top to bottom at a 90-degree angle. The wells were washed twice with PBS. KGM, KGMwo.s. and serum samples in KGMwo.s. were added. KGM served as a positive control and KGMwo.s. as a negative control. KGMwo.s. was made of KGM medium without the addition of the following supplements: bovine pituitary extract, h-EGF, insulin and hydrocortisone.

**Figure 12:**
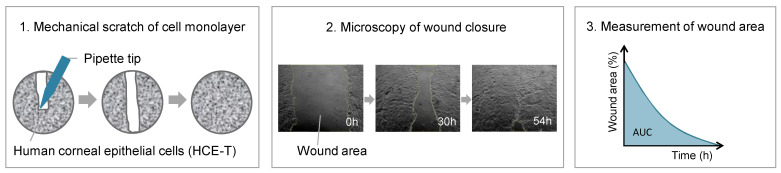
Procedure of the DED wound healing model.

Cells were analyzed under an Olympus IX50 microscope (Olympus, Hamburg, Germany) with a 4-fold objective until wound closure or at maximum for five days. Six pictures per well were taken. A starting point was marked with a felt-tip pen underneath the well to analyze the same area every time. Wound areas were measured using Fiji software [72]. The results are depicted as the percentage of wound area over time using Origin(Pro) (Version 2019b, OriginLab Corporation, Northampton, MA, USA). The procedure of the wound healing model is shown in Figure 12. The wound healing model experiments are described in the following paragraphs.

#### 4.3.1. Assay Development

##### Influence of Coating Materials

The influence of different coating materials on the wound closure rate was analyzed. The coating materials used were picked due to their appearance in the extracellular matrix of corneal cells [73]. First, collagen type I was tested using a concentration of 1.5 mg/mL in ethanol. Then, collagen was combined with fibronectin (10 µg/mL). In a third step, collagen, fibronectin and laminin (10 µg/mL) were used. The coating materials were applied on the day prior to seeding the HCE-T cells. HCE-T cells were cultivated with KGM medium during the experiment.

##### Serum Concentration

The following serum concentrations were analyzed in the wound healing model: 1%, 2.5%, 5%, 10%, 20%, 30% 50% and 100% allogeneic serum. Serum concentrations of 20% and 50% are often used in clinical research [4,5,74]. The lower concentrations were added to find a model that could differ between working batches of serum and noneffective serum batches.

##### Intralaboratory Variance

First, it was analyzed whether or not the wound healing model could differentiate between positive and negative controls. This functionality test was conducted with the help of an independent *t* test. Therefore, the AUC of the wound healing curve (Figure 1) of the positive and negative controls was compared between two researchers. Second, an equivalent test was conducted to test the intralaboratory variance of the wound healing model. The 95% confidence interval of the AUC from KGM and KGMwo.s. from researcher one was determined for all nine independent experiments using the following equation:

(1) $$\mathrm{CI}=\bar{x}\pm 1.96\times \sigma \times \sqrt{n}$$

(CI = confidence interval; x = mean; *n* = sample size; σ = standard deviation). The percentage-based deviation based on the greatest confidence interval of the AUC of KGM and KGMwo.s. of researcher one served as upper and lower limits for the equivalence test. The 95% confidence intervals from researcher two were tested to see if they would fit into the set limits determined by the greatest deviation of the CI of researcher one.

#### 4.3.2. Preclinical Applications

##### Stability Assay

The DED wound healing assay served as a method to analyze serum stability. Serum samples stored at −20 °C over four different time periods were analyzed in the DED wound healing model. The following time points were used: 24 h, one month, three months and six months. The serum of two different donors from two serum batches was examined. The serum concentration used was 2.5%.

##### Donor Variability

The DED wound healing assay was used to test whether different blood donors had an influence on the wound-healing potential of serum. Hence, allogeneic serum samples from five different donors were analyzed in the DED wound healing model. The serum concentration used was 1%.

##### Influence of Freeze–Thaw Cycles

Serum undergoing several freeze–thaw cycles was tested in the wound healing model. One, two, five and ten cycles with at least 30 min at −20 °C and at least 20 min at room temperature were tested. The serum concentration used was 2.5%.

### 4.4. Inflammatory DED Model

#### 4.4.1. Static Inflammatory DED Model

HCE-T cells were seeded into 6-well plates (250,000 cells per well) and cultivated in KGM medium for nine days. On day ten, the cells were transferred to KGMwo.s. medium. Subsequently, 10 ng/mL TNFα or 10 ng/mL IL-1β was added to the medium for 24 h. First, the gene expression of MMP9, COX-2, TGFβ and IL-1β was tested with and without serum 1%. Dexamethasone (100 µM) served as a positive control. HCE-T cells in KGMwo.s. medium served as a negative control. Second, the following serum concentrations were added simultaneously and tested for their influence on the gene expression of MMP9 after 10 ng/mL TNFα induction: 1%, 2.5%, 5%, 10%, 20%, and 50%. Here, KGMwo.s. medium served as interrun calibration. After incubation with cytokines, RNA was isolated, cDNA was synthesized, and qPCR was carried out.

##### RNA Isolation

RNA was isolated using TRIzol reagent. After five minutes at room temperature, chloroform was added, and the vials were shaken and incubated for three minutes at room temperature. The vials were then centrifuged (Allegra 64R Centrifuge, Beckman Coulter, Brea, CA, USA) at 4 °C for 15 min at 12,000× *g*. The upper phase was extracted from the vial, and isopropanol was added. After 10 min at room temperature, the samples were centrifuged again at 4 °C at 12,000× *g* for 10 min. The supernatant was discarded, and the RNA pellet was washed twice with 75% ethanol diluted with diethyl pyrocarbonate (DEPC) water. Then, the pellet was centrifuged again at 7500× *g* and 4 °C for five minutes. The ethanol supernatant was discarded, and the pellet was resuspended in DEPC water. The samples were dissolved at 58 °C for 10 min in a thermal block (Eppendorf, Hamburg, Germany). RNA concentration, A 260/230 and A 280/260 values were detected using the Nanoquant plate (Tecan, Männedorf, Switzerland) and the Tecan GENios microplate reader (Infinite M Plex, Tecan). Samples were stored at −80 °C until cDNA synthesis.

##### cDNA Synthesis

One microgram of RNA was used for cDNA synthesis. cDNA was obtained using the iScript gDNA Clear cDNA Synthesis Kit. A nonreverse transcriptase control (NRTK) was used with the cDNA of the control sample. cDNA samples were stored at −20 °C until further usage for qPCR.

##### qPCR

qPCR was carried out using the CFX Connect Real-time PCR Detection System (Bio-Rad, Hercules, CA, USA). The reagent dye universal SYBR Green Supermix was used. Probes were diluted with DEPC water. Glyceraldehyde-3-phosphate dehydrogenase (GAPDH) and β-actin were used as housekeeping genes. The following target genes were analyzed: MMP9, COX2, TGFβ and IL-1β. The primer sequences for GAPDH were used as described by Kim et al. [45], and the primer sequences for β-actin and MMP9 were used as described by Lu et al. [30]. Primer sequences for COX2, TGFβ and IL-1β were designed using Primer-BLAST software [75]. Primer secondary structures were analyzed by Beacon Designer^TM^ Free Edition software (PREMIER Biosoft, San Francisco, CA, USA) for self-designed primers. Product secondary structures were analyzed by mFold software [76]. Gene sequences of forward and reverse primers and the annealing temperature of the analyzed genes as well as their efficiency are depicted in Table 2.

Primer efficiency was analyzed via qPCR, and the optimal annealing temperature (T_A_) was generated using a temperature gradient between 50 and 58 °C. The relative normalized gene expression (ΔΔCq) was normalized to the KGMwo.s. sample as a negative control and normalized to the housekeeping genes. Gene expression was calculated via the Pfaffl method with CFX Maestro software (Bio-Rad, Hercules, CA, USA).

#### 4.4.2. Dynamic Inflammatory DED Model

**Figure 13:**
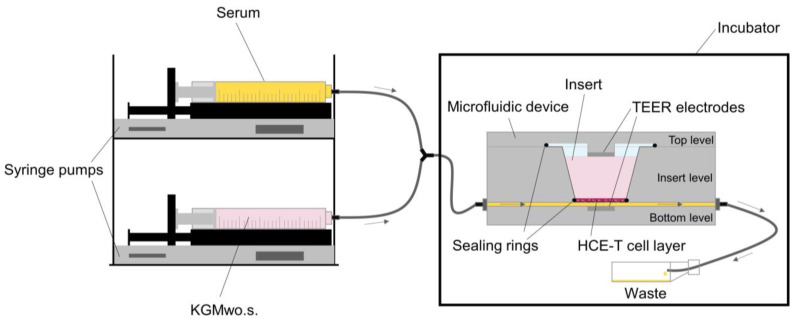
Microfluidic device (Ocular DynaMiTES) for the dynamic inflammatory DED model.

A total of 100,000 HCE-T cells were seeded into Transwell^®^ inserts and cultivated for eight days. On day nine, 10 ng/mL TNFα and 10 ng/mL IL-1β in KGMwo.s. were added, and HCE-T cells were cultivated for another 72 h. After incubation, the medium with cytokines was removed, and the cells were transferred into new KGMwo.s. medium. The inserts were transferred to the microfluidic Ocular DynaMiTES system as described by Lorenz et al. [77]. Ocular DynaMiTES is a microfluidic system linked to two syringe pumps to mimic continuous tear film fluidics (Figure 13).

It is placed in a cell culture incubator and can be programmed for different flow rates. Two electrodes measure TEER values (transepithelial electrical resistance) continuously as internal control values of the cellular barrier properties. Serum was added with a turnover rate of 8.3% as previously described by Lorenz et al. [77]. Moreover, a turnover rate of 8.3% was measured in patients during the basal phase of tear production [78]. The additional serum was continuously diluted with KGMwo.s. medium in the second syringe pump. A flow rate of 75 µL/min was used. The system was run for 180 minutes. One model was treated with serum under the set dilution rates using TNFα- and IL-1β-treated cells. A second model was run with KGMwo.s. medium using induced cells. A third model was run with noninduced cells cultivated in KGMwo.s. medium. After finishing the DynaMiTES run, the cells were transferred to TRIzol reagent and stored at −80 °C until RNA isolation. Then, cDNA synthesis and qPCR were carried out to examine MMP9 expression.

#### 4.4.3. Design of Experiments

For the DoE studies, HCE-T cells were cultivated for nine days in 6-well plates (250,000 cells per well) in KGM medium. The cells were induced on day ten with different TNFα and IL-1β concentrations for different periods following the experimental design. Cornerstone Software (Version 7.1.0.3, CamLine, Petershausen, Germany) was used both for experimental design and regression analysis. The TNFα and IL-1β (0–20 ng/mL) concentrations as well as the incubation time (0 h–48 h) were used as continuous factors for the model. The relative normalized gene expression of MMP9 in HCE-T cells was used as a metric in the model. The aim was to maximize the gene expression with an estimated target value of 50. A modified D-optimal design was used to add two-hour timepoints as inclusions in the model and higher order terms for the middle of the design space. Furthermore, the following constraints were added to the model: time should not be zero, and the sum of IL-1β and TNFα concentrations should not be zero. The 21 runs calculated by the software are depicted in Table 3.

#### 4.4.4. Inflammatory DED Protein Expression Model

MMP9 protein expression was analyzed with the Human MMP-9 DuoSet ELISA following the manufacturer’s protocol. HCE-T cells were seeded into 6-well plates (250,000 cells per well) and cultivated for ten days. HCE-T cells were induced with TNFα (10 ng/mL) for 24 h, and simultaneously, 1%, 2.5%, 5%, 10%, 20% and 50% human allogeneic serum was added. Dexamethasone (100 µM) served as a positive control. HCE-T-cell supernatants were obtained and stored at −20 °C until use. Serum samples diluted with reagent diluent served as a positive control. The values of the positive controls were subtracted from the sample values. Absorbance was measured at 450 nm, and the reference wavelength was 540 nm using a microplate reader (Infinite M Plex, Tecan, Männedorf, Switzerland). The standard curve of the standard dilution series was exponentially fitted using Origin(Pro) (Version 2019b, OriginLab Corporation, Northampton, MA, USA).

### 4.5. Statistical Analysis

Statistical analysis was performed using a one-way ANOVA with a Games–Howell or Bonferroni post hoc test (depending on the homogeneity of the variances) with IBM^®^ SPSS^®^ statistics (Version 28.0, IBM, Armonk, NY, USA). Data of the intralaboratory variance were analyzed statistically using an independent *t* test. Statistical significance was assumed with *p* ≤ 0.05 (one asterisk). *p* ≤ 0.01 is depicted with two asterisks, and *p* ≤ 0.001 is depicted with three asterisks. Data were calculated as the means and standard deviation or as the standard error of the mean in the DoE experiment.

## 5. Conclusions

In conclusion, the two developed in vitro DED models can be used to examine wound healing and anti-inflammatory effects of human allogeneic serum eye drops in preclinical studies. The DED wound healing model is helpful to study the stability and donor variability of different serum batches. Additionally, the optimized inflammatory DED model is suitable to show anti-inflammatory effects of allogeneic serum under static and dynamic flow conditions in preclinical assays. Hence, the developed DED models present an effective tool to analyze allogeneic serum and other substances for DED treatment in prospective preclinical studies. To confirm the wound healing and anti-inflammatory effects of allogeneic serum, in vivo studies should be performed in the future.

## Figures and Tables

**Table 1:** MMP9 model coefficient table depicting the used terms of the model with their coefficient value, standard error, t value and significance level; italic terms are not significant but remain in the model.

Term	Coefficient	Std Error	T Value	Significance
*Constant*	−14.2223	7.6350	−1.8628	0.0995
TNFα	−4.5901	1.8952	−2.4220	0.0417
*IL-1β*	0.9070	0.7094	1.2785	0.2369
Time	8.8916	1.7292	5.1419	0.0009
IL-1β * TNFα	0.0588	0.0225	2.6062	0.0313
TNFα * Time	0.0829	0.0103	8.0365	0.0000
IL-1β * Time	0.0459	0.0101	4.5253	0.0019
TNFα^2^	0.6988	0.2451	2.8517	0.0214
IL-1β^2^	−0.0971	0.0268	−3.6186	0.0068
Time^2^	−0.5878	0.1498	−3.9239	0.0044
Time^3^	0.0151	0.0046	3.2852	0.0111
TNFα^3^	−0.0268	0.0081	−3.3247	0.0105
Time^4^	−0.0001	0.0000	−2.9901	0.0173

**Table 2:** Primer sequences of housekeeping and inflammatory genes and their annealing temperatures and efficiencies.

Gene	Sequence	TA (°C)	Efficiency
GAPDH	F GAA GGT GAA GGT CGG AGT R GAA GAT GGT GAT GGG ATT TC	53.2	99.8%
β-actin	F GCT ATT TGG CGC TGG ACT T R GCG GCT CGT AGC TCT TCT C	53.2	108.6%
MMP9	F AGT ACC GAG AGA AAG CCT ACT T R TGC AGG ATG TCA AAG CTC AC	50.5	94.2%
COX2	F GAC GCC CTC AGA CAG CAA AG R TGG GTG GGA ACA GCA AGG ATT	58.5	93.0%
TGFβ	F ATT CCT GGC GAT ACC TCA GCA R TGA ACC CGT TGA TGT CCA CTT G	58.5	98.8%
IL-1β	F TCT TCG AGG CAC AAG GCA CA R GCC ATC ATT TCA CTG GCG AGC	58.5	116.2%

**Table 3:** Experimental design of the design-of-experiments gene expression model.

Run	TNFα (ng/mL)	IL-1β (ng/mL)	Time (h)
1	7	13	36
2	7	0	24
3	20	20	12
4	0	20	24
5	13	13	48
6	20	0	36
7	0	7	36
8	0	10	2
9	20	13	24
10	7	7	12
11	20	0	12
12	13	0	24
13	20	0	48
14	20	20	48
15	13	20	36
16	10	0	2
17	0	13	12
18	7	0	48
19	20	7	24
20	10	10	2
21	0	20	48

## Data Availability

Data available upon reasonable request from corresponding author.
